# Granular Cell Tumor in a 13-Year-Old Girl

**DOI:** 10.7759/cureus.19199

**Published:** 2021-11-02

**Authors:** Amy L Fraser, Benjamin P Anthony

**Affiliations:** 1 Otolaryngology, Indiana University School of Medicine, Indianapolis, USA

**Keywords:** laryngoscopy, laryngeal lesion, pediatric, dysphonia, granular cell tumor

## Abstract

Granular cell tumors are rare benign soft-tissue lesions that most commonly occur in the head and neck. They usually present in adulthood and are rarely seen in children. Here we present a 13-year-old girl who experienced symptoms of hoarseness of voice for most of her childhood and was unsuccessfully treated for asthma, acid reflux, allergies, and bronchitis before direct visualization revealed what was initially thought to be a vocal cord cyst. Surgical excision and pathology revealed the unexpected diagnosis of a vocal cord granular cell tumor. The patient has had resolution of dysphonia and is undergoing voice therapy.

## Introduction

Granular cell tumors (GCTs) are rare neoplasms that most commonly affect the skin and subcutaneous tissue [1-3], with approximately half found in the head and neck region. While the tongue is commonly involved, only 6% to 10% of head and neck cases occur in the larynx [4]. The majority of GCTs are benign, while malignant GCTs are extremely rare and comprise less than 2% of cases [1]. Though originally thought to be of muscular origin [2,5], immunohistochemical studies demonstrating positive staining for S100 protein, neuron-specific enolase, and myelin-specific protein indicate they are Schwann cell derivatives [6]. They have a very characteristic appearance under the microscope [7], and can often be diagnosed with routine hematoxylin-eosin-stained sections, though immunohistochemical staining helps confirm the diagnosis [6]. 

GCTs can affect any age group, but are most commonly present during the fourth to sixth decade of life [1,2,5] and may be more common in women [1,4,5]. They are rarely seen in children, with only 38 pediatric cases of laryngeal GCTs in the literature [4]. Laryngeal GCTs present similarly to other laryngeal lesions, with hoarseness, stridor, dysphagia, and occasional hemoptysis, and patients may be misdiagnosed with asthma or bronchitis [4,8]. Here we present a case of a pediatric patient with longstanding hoarseness.

## Case presentation

A 13-year-old girl presented for evaluation of hoarseness. The patient’s mother noted that the patient had always spoken with a hoarse voice and that the hoarseness had worsened in the past five years. During this time she attempted treatments for asthma, acid reflux, allergies, and bronchitis, with no improvement of hoarseness. The patient had not had a formal otolaryngology workup before now. The patient denied sore throat, dysphagia, or globus sensation. She had no difficulty swallowing and denied postnasal drip or congestion. She has no chronic medical conditions. She underwent tonsillectomy and adenoidectomy three years ago due to recurrent infections. She is otherwise healthy with normal development.

Her voice was mostly rough per GRBAS scale (Grade 2, Roughness 2, Breathiness 0, Asthenia 0, Strain 1). She underwent rigid videostroboscopy which revealed full vocal fold mobility bilaterally, large left-sided infraglotttic swelling of the entire length of the vocal fold, with a rounded swelling at the midmembranous area. Glottic closure was complete with no lesions in the vallecula, larynx, or hypolarynx. Mucosal wave propagation showed chasing wave asymmetry with decreased amplitude on the left but normal on the right. There were no signs of reflux, the trachea was widely patent, and she displayed no gross aspiration. A presumed diagnosis of vocal fold cyst was made.

**Figure 1:**
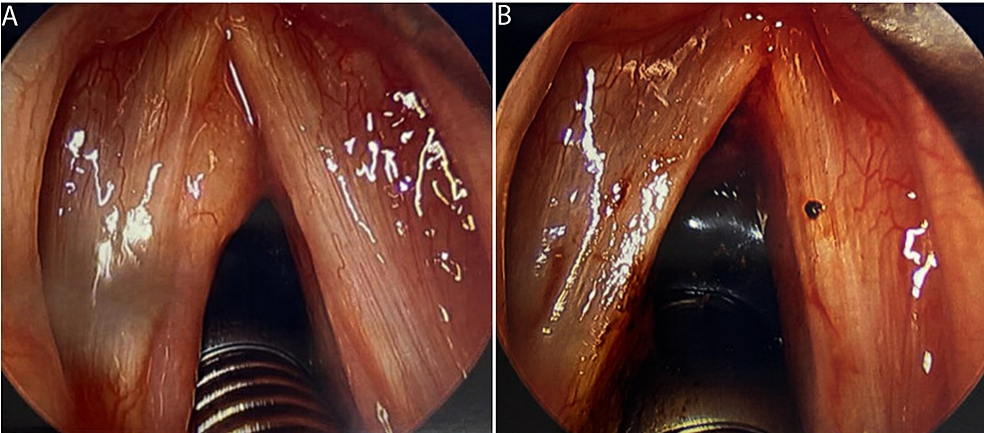
Visualization of the true vocal folds. A: Pre-operative findings are a firm, fibrous submucosal mass on the left extending the entire length of the vocal fold, primarily anteriorly. B: Post-operative view of the true vocal folds.

Following induction of general endotracheal anesthesia, a size 4 Zeitels laryngoscope was inserted into the patient’s mouth and the larynx was visualized (Figure 1 A). A firm, fibrous submucosal mass was found extending the entire length of the left vocal fold with an anterior predominance. Next, an epithelial cordotomy was made on the superior lateral surface of the left vocal fold the entire length of the lesion. The superficial layer of the lamina propria was gently dissected away from the lesion and the lesion was fully demarcated. Using microlaryngeal scissors and forceps the lesion was removed and sent for pathologic evaluation. Redundant mucosa was trimmed, and a mucosal flap was used to cover the defect. A CO_2_ laser was brought in to remove any protrusion of the vocal fold to give a straight phonating edge (Figure 1 B). An injection of 0.3 mL of injectable implant was performed to prevent glottic insufficiency.

**Figure 2:**
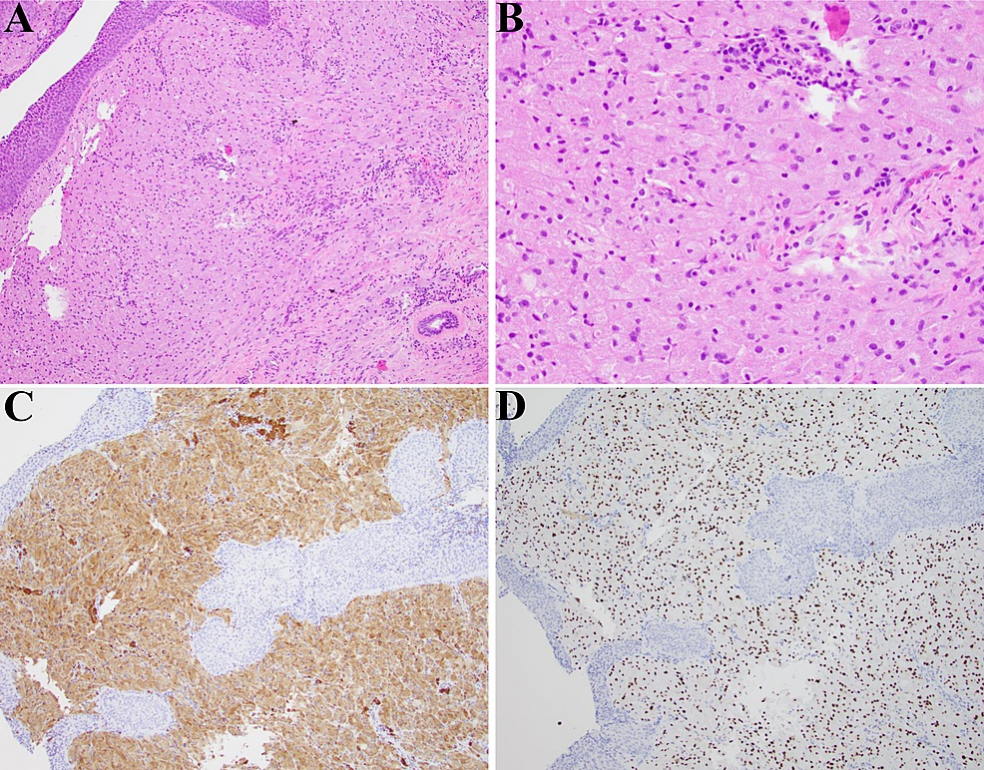
A: respiratory mucosa with a lesion in the superficial submucosa (H&E 100X magnification). B: The lesion consists of large histiocytoid cells with abundant granular cytoplasm and oval shaped nuclei with inconspicuous nucleoli (H&E 200X magnification). C: CD68 immunostain is strongly and uniformly positive in the tumor cells with a cytoplasmic pattern. D: SOX10 immunostain is strongly and uniformly positive in the tumor cells with a nuclear pattern. H&E: Hematoxylin-eosin; SOX10: SRY-box transcription factor 10; CD68L: Cluster of differentiation 68

The specimen consisting of three irregular, gray-tan soft tissues with the greatest dimensions of 0.2 cm, 0.3 cm, and 0.6 cm were submitted for pathological evaluation. Hematoxylin and eosin stain revealed large cells with abundant granular cytoplasm (Figure 2 A and B). Immunostaining was positive for cluster of differentiation 68 (CD68) as seen in Figure 2 C, SRY-box transcription factor 10 (SOX10) in Figure 2 D, and S100 (not pictured). The specimen was negative for desmin, smooth muscle antibody (SMA), and AE1/AE3. The final diagnosis was a granular cell tumor of the left true vocal fold. The patient reported throat tenderness during the first few postoperative days but on the one-week follow-up reported improvement and reduced hoarseness. She is continuing to be followed and is due to begin voice therapy.

## Discussion

Granular cell tumors are rare lesions of Schwann cell origin that may arise anywhere in the body and are often found in the head and neck region [4,5]. They are rarely found in children. In their review, Mur et al. found only 15 instances of pediatric laryngeal GCTs affecting the true vocal folds [4]. In the larynx, the most common presenting symptom is dysphonia (81%), followed by stridor (23%) and dyspnea (23%). In a few cases, patients have reported cough and exercise intolerance [4].

Granular cell tumors typically appear as flesh-colored or reddish-brown, solitary, painless, slow-growing nodules that are less than 3 cm to 4 cm in diameter [5]. On the true vocal cords it is difficult to differentiate GCTs from polyps, granulomas [3], or cysts by direct visualization alone. Because of this, diagnosis generally relies on excision and pathological examination. They have a very characteristic appearance with hematoxylin-eosin staining showing abundant eosinophilic, granular cytoplasm, but immunohistochemical staining may be required to confirm diagnosis. This is especially true in cases where there is marked pseudoepitheliomatous hyperplasia, which can mimic squamous cell carcinoma and other neoplasms with abundant eosinophilic granular cytoplasm [6]. As in our case, SOX100, CD68, and S100 are generally positive [6] and point to GCTs of Schwann cell origins.

Granular cell tumors generally present as single lesions, but the presence of multiple lesions should raise the suspicion for syndromes with which they are associated, such as Noonan syndrome, neurofibromatosis type I, and LEOPARD syndrome (Lentigines, Electrocardiographic conduction defects, Ocular hypertelorism, Pulmonary stenosis, Abnormalities of the genitalia, Retarded (slowed) growth, Deafness) [1,5]. While the majority of cases are benign, 1% to 2% of cases behave malignantly. Malignant lesions are generally larger than benign lesions, and often present with metastases to lymph nodes, lung, and bones. They exhibit rapid growth, ulceration, and invasion of local structures. These cases often have a poor prognosis with limited curative options. Radiotherapy and chemotherapy are rarely affective [5,9]. 

Treatment is complete surgical excision, and regular follow-up is required as recurrence is known to occur even in benign lesions. For benign lesions, complete surgical excision is considered curative, with a recurrence rate between 2% to 8% [10]. Incomplete excision may lead to local recurrence in 21% of cases [2]. Though there is no standard algorithm for post-treatment surveillance, long-term follow-up via direct visualization is necessary to ensure there is no recurrence, and to monitor for multifocal disease [4].

## Conclusions

Granular cell tumors are a rare cause of dysphonia in children. Patients are often unsuccessfully treated for bronchitis or asthma for years before accurate diagnosis and treatment. Visualization of the tumor may be inconclusive as it closely resembles more common vocal cord masses and biopsy is often required for definitive diagnosis. Treatment is surgical, and curative in most patients, though speech or voice therapy may be beneficial in some cases, and regular long-term follow-up is recommended.
